# Synergistic strategies for high production of *Geobacillus stearothermophilus* α-amylase in *Bacillus subtilis*

**DOI:** 10.1093/jimb/kuaf036

**Published:** 2025-12-19

**Authors:** Deming Rao, Changhui Wang, Xiaolin Li, Wei Shen, Qiang Liu, Zerui Li, Shiyu Pi, Zhenggang Han, Jiangke Yang

**Affiliations:** School of Life Science and Technology, Wuhan Polytechnic University, Wuhan 430023, People’s Republic of China; School of Life Science and Technology, Wuhan Polytechnic University, Wuhan 430023, People’s Republic of China; Tianjin Institute for Drug Control (TIDC), 98 Guizhou Road, Tianjin 300070, PR China; School of Life Science and Technology, Wuhan Polytechnic University, Wuhan 430023, People’s Republic of China; School of Life Science and Technology, Wuhan Polytechnic University, Wuhan 430023, People’s Republic of China; School of Life Science and Technology, Wuhan Polytechnic University, Wuhan 430023, People’s Republic of China; School of Life Science and Technology, Wuhan Polytechnic University, Wuhan 430023, People’s Republic of China; School of Life Science and Technology, Wuhan Polytechnic University, Wuhan 430023, People’s Republic of China; School of Life Science and Technology, Wuhan Polytechnic University, Wuhan 430023, People’s Republic of China

**Keywords:** α-amylase, *Bacillus subtilis*, 5-L bioreactor

## Abstract

Although the α-amylase (AmyS) from *Geobacillus stearothermophilus* exhibits high thermostability, its low enzymatic activity (44.36 ± 2.02 U/mL) severely hinders industrial applications. Given its strong protein secretion capability and GRAS (generally recognized as safe) status, this study employed *Bacillus subtilis* as the host to enhance AmyS production. Through error-prone PCR and high-throughput screening, a triple mutant T151A/K178E/T458A (AmySM) was generated, showing a 17.67% increase in activity (51.34 ± 1.11 U/mL). AmySM activity was further increased by 29.32% to 65.23 ± 2.33 U/mL using the signal peptide SP*_ykwD_*, selected from a comprehensive library for superior secretion efficiency. The promoter P*_gsiB_* enhanced activity by 37.61% to 89.54 ± 2.95 U/mL. Optimizing the ribosome binding site (RBS) resulted in an additional 48.83% increase in activity, yielding a final activity of 134.02 ± 3.54 U/mL, which corresponds to a 3.02-fold improvement over the initial strain WBSW (44.36 ± 2.02 U/mL). Ultimately, scale-up fermentation in a 5-L bioreactor yielded a maximum extracellular activity of 1244.17 ± 48.66 U/mL at 72 hours, a 2.84-fold increase over the control. This multi-level strategy provides a rational framework for high-efficiency AmySM production, paving the way for extracellular production of high-value proteins in the GRAS host *B. subtilis*.

## Graphical Abstract

**Graphical Abstract:**
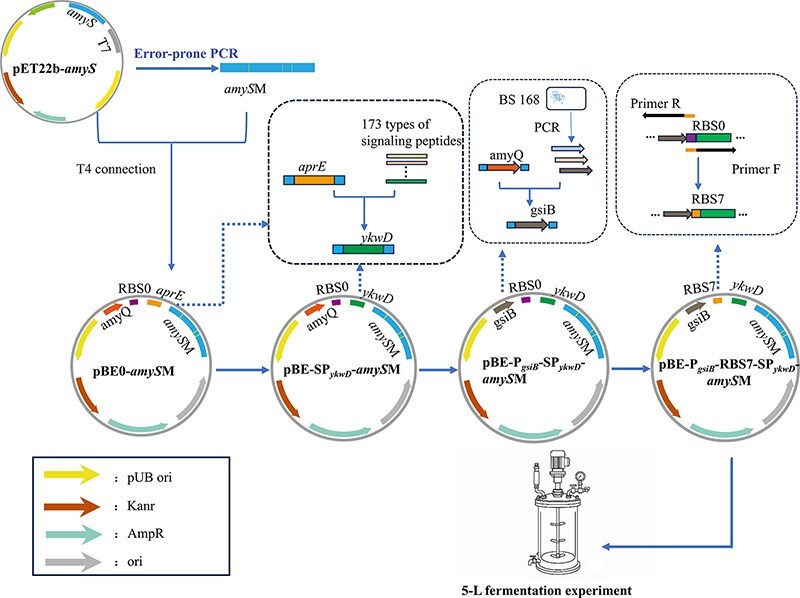
This study uses a multi-level strategy to significantly enhance the activity of AmyS in the GRAS host *Bacillus subtilis*, achieving high extracellular production.

## Introduction

Alpha (α)-amylases (EC 3.2.1.1) are glycoside hydrolases that catalyze the hydrolysis of internal α-1,4-glycosidic bonds in starch and related polysaccharides, yielding low molecular weight sugars including dextrins, maltose, maltooligosaccharides, and glucose (Burhanoğlu et al., 2020). α-Amylases are among the most important industrial enzymes, comprising ∼30% of the global enzyme market (Paul et al., 2021). Those from *G. stearothermophilus* are particularly valued for their high catalytic efficiency and are widely used in food, fermentation, textiles, detergent and biofuel production (Hu et al., 2023; Li et al., 2022b).

Although many amylases have been discovered, only few exhibit high specific activity toward starches. This has driven the need for adaptive modification techniques, such as directed evolution and immobilization, to improve catalytic performance. Directed evolution, unlike natural evolution, is an artificial approach designed to rapidly generate enzyme variants with desired traits via repeated cycles of mutation and screening (Boersma et al., 2008; Zeng et al., 2020). Yao et al. employed error-prone PCR and high-throughput screening in *B. subtilis*, yielding a mutant variant (AmySA), with a 1.44-fold enhanced activity compared to the wild-type (Yao et al., 2019). Similarly, You et al. and Shi et al. engineered the subtilisin E mutant 13 M and the lignin peroxidase mutant N120D/I242T, which yielded 16-fold and 196.36% higher activity, respectively (Shi et al., 2024; You and Arnold, 1996). Collectively, these findings underscore the broad potential of directed evolution for optimizing enzymes in *B. subtilis*, exploiting its high protein secretion capacity.

Besides catalytic efficiency, the yield of recombinant α-amylases is another major bottleneck for its industrial applications. *Bacillus subtilis* emerges as a premier host to address this constraint, owing to its exceptional protein secretion capacity and high genetic tractability (Li et al., 2022b; Yang et al., 2021). Several strategies have been developed to enhance the protein expression capability of *B. subtilis*, such as signal peptides screening, promoter replacement, and ribosome binding site (RBS) optimization (Yang et al., 2020).

The signal peptide (SP) plays a pivotal role in directing proteins to the secretion machinery, thereby determining both expression level and extracellular yield (Liu et al., 2019a; Tjalsma et al., 2000). Common strategies for signal peptide (SP) optimization encompass rational design and empirical screening. Rational approaches include site-directed or random mutagenesis, modifying charged residues in the N-region, and adjusting the distance between the SP and the mature protein. However, high-throughput screening of homologous or heterologous signal peptide libraries has proven particularly powerful and extensively employed to enhance heterologous protein secretion efficiency in *B. subtilis* and other expression systems (Fu et al., 2018; Khadye et al., 2021). By screening a library of 173 native signal peptides (SPs) from the *B. subtilis* 168 genome, Yao et al. identified SP*_yojL_* as the optimal sequence, achieving a 3.5-fold enhancement in α-amylase secretion over the control (Yao et al., 2019). Fu et al. built a library comprising 173 native Sec-type SPs. Of these, SP*_pel_* conferred the highest secretion efficiency, which culminated in a maximum α-amylase activity of 5086 U/mL during high-cell-density fermentation (Fu et al., 2018).

Transcriptional regulation, primarily governed by the promoter, is a key determinant of recombinant protein yield. The promoter’s strength directly controls the efficiency of transcript synthesis (Öztürk et al., 2017). Given the cost advantage, constitutive and auto-inducible promoters highly attractive for industry. Consequently, promoter screening has been successfully implemented in *B. subtilis* to significantly boost the extracellular expression of recombinant proteins. Through engineering of the P*_spoVG_* promoter, Li et al. achieved a 2.05-fold increase in Amyz1 production, reaching a high titer of 952.6 U/mL (Li et al., 2022a). By employing a sigma factor-specific promoter screening strategy, Kang et al. found that P*_amyE_* enhanced amidase production by 1.46-fold, reaching a yield of 55.19 U/mL compared to the control strain (Kang et al., 2020).

At the translational level, the ribosome binding site (RBS) play a vital role by controlling the efficiency of translation initiation (You et al., 2024). Unlike *Escherichia coli*, the native RBS in *B. subtilis* is often suboptimal for heterologous gene expression. This inherent limitation makes RBS engineering essential for maximizing protein synthesis (Rao et al., 2024). Rational RBS optimization is a powerful strategy to boost target protein expression. By predictably fine-tuning the translation initiation efficiency, it directly increases protein synthesis yields. By optimizing the translation initiation rate, Li et al. significantly enhanced L-asparaginase production, achieving an activity of 371.87 U/mL, a 2.09-fold increase over the control strain (Li et al., 2019). Using the RBS prediction tool from the Salis Lab (https://salislab.net/software/), Pang et al. rationally designed a sequence that optimized the translation initiation rate of pullulanase gene. This strategy successfully increased extracellular enzyme activity to 118 U/mL, which is 1.9 times higher over the control strain (Pang et al., 2020).

Various expression systems for *Geobacillus stearothermophilus* α-amylase have been explored. For instance, Ozdemir et al. optimized fermentation parameters for the wild-type strain *G. stearothermophilus* ATCC 12980 but achieved a low α-amylase activity of only 0.95 ± 0.16 U/mL (ÇAliŞKan ÖZdemİR et al., 2016). In contrast, a marked improvement was achieved through recombinant expression. By expressing the *amyS* from *G. stearothermophilus* US100 in *E.coli*, Khemakhem et al. reported a final α-amylase activity of 2690 U/mL, thereby demonstrating the high-yield potential of this heterologous system (Khemakhem et al., 2009). Using the methylotrophic yeast *Pichia pastoris* GS115 as a host, Gandhi et al. expressed of the *amyS* from *G. stearothermophilus* SR74. Methanol-induced fermentation yielded a recombinant enzyme activity of 28.6 U/mL (Gandhi et al., 2015). These findings collectively demonstrate that the choice of host system is critical for the production efficiency and fnal yield of recombinant α-amylase. Given this significant impact, it is noteworthy that the *amyS* from *G. stearothermophilus* has not been expressed in *B. subtilis*-a well-established, food-grade host with excellent potential for industrial enzyme production. Therefore, the establishment of such an *amyS* expression system in *B. subtilis* would constitute a significant advance, both as a robust scientific platform and for its direct applications in food-grade industrial enzyme production.

In this study, we implemented a comprehensive engineering strategy in *B. subtilis* WB600 to enhance the extracellular production of *G. stearothermophilus* α-amylase (AmyS). Our multipronged approach encompassed directed evolution to improve catalytic activity, high-throughput signal peptide screening to facilitate secretion, promoter screening to augment transcriptional levels, and RBS optimization to optimize translational efficiency. The integrated engineering strategy dramatically enhanced extracellular enzyme activity at both shake-flask and 5-L fermenter scales. The study establishes a versatile and modular framework for recombinant enzymes production in *B. subtilis*, with strong potential for industrial biomanufacturing applications.

## Materials and Methods

### Chemicals, Reagents and Culture Medium

All restriction endonucleases were purchased from Takara. T5 exonuclease was sourced from New England Biolabs (NEB). Tryptone and yeast extract were procured from Oxoid. Isopropyl β-D-thiogalactoside (IPTG) was supplied by Sigma-Aldrich. Other chemicals were acquired from Sangon Biotech, China. Luria-Bertani (LB) medium consisted of 10 g/L tryptone, 10 g/L sodium chloride, and 5 g/L yeast extract. For solid LB medium, 15 g/L agar powder was supplemented to the liquid LB. The TB medium was formulated with 12 g/L tryptone, 24 g/L yeast extract, 6.3 g/L glycerol, 2.3 g/L KH_2_PO_4_ and 12.5 g/L K_2_HPO_4_. The medium used in the 5-L fermenter included 15 g/L tryptone, 15 g/L yeast extract, 12 g/L glucose, 1 g/L MgSO_4_·7H_2_O, 1 g/L (NH4)_2_-H-citrate, 2.68 g/L (NH4)_2_SO_4_, 2 g/L Na_2_SO_3_, 4 g/L NaH_2_PO_4_·H_2_O, 14.6 g/L K_2_HPO_4_, and 3 mL trace element solution. The feeding medium for the 5-L fermenter consisted of 24 g/L tryptone, 48 g/L yeast extract, 100 g/L glucose, and 40 mL trace element solution. The trace element solution was formulated with 0.1 g/L MnSO_4_·H_2_O, 0.18 g/L CoCl_2_·6H_2_O, 0.16 g/L CuSO_4_·5H_2_O, 0.18 g/L ZnSO_4_·7H_2_O, 0.5 g/L CaCl_2_, 8.35 g/L FeCl_3_, and 10.05 g/L Na_2_EDTA.

### Strains and Plasmids

All strains and plasmids used in this study are listed in Table 1. *E. coli* DH5α was used as a host strain for plasmid construction. *E. coli* BL21 and *B. subtilis* WB600 were used for recombinant expression of *amyS* and *amyS*M. The PCR reaction mixture for recombinant plasmid construction comprised 25 μL of 2 × KeyPo Master Mix, 2 μL of forward primer (10 μM), 2 μL of reverse primer (10 μM), 2 μL template DNA (5–10 ng), with nuclease-free water added to a final volume of 50 μL. Primer sequences are listed in Table S1. Recombinant plasmids were electroporated into *B. subtilis* WB600 competent cells to construct engineered expression strains, following the method of Anagnostopoulos (Anagnostopoulos and Spizizen, 1961).

**Table 1.** Strains and plasmids

Strains or plasmids	Description	Reference
Strains		
*E. coli* DH5α	Clone strain	Takara
*E. coli* BL21	Protein expression host	Our laboratory
*B. Subtilis* WB600	*B. subtilis* 168 derivate, deficient in nprE, aprE, epr, bpr, mpr, nprB, expression strain	Our laboratory
WBSW	WB600/pBE-P*_amyQ_*-RBS0-SP*_aprE_*-*amyS*	This work
WBK	WB600/pBE-P*_amyQ_*-RBS0-SP*_aprE_*	This work
WBSM	WB600/pBE-P*_amyQ_*-RBS0-SP*_aprE_*-*amyS*M	This work
WBSM1	WB600/pBE-P*_amyQ_*-RBS0-SP*_aprE_*-*amyS*M1	This work
WBSM2	WB600/pBE-P*_amyQ_*-RBS0-SP*_aprE_*-*amyS*M2	This work
WBYdjM	WB600/pBE-P*_amyQ_*-RBS0-SP*_ydjM_*-*amyS*M	This work
WBYqxL	WB600/pBE-P*_amyQ_*-RBS0-SP*_yqxL_*-*amyS*M	This work
WBAbnAw	WB600/pBE-P*_amyQ_*-RBS0-SP*_abnAw_*-*amyS*M	This work
WBYkwD	WB600/pBE-P*_amyQ_*-RBS0-SP*_ykwD_*-*amyS*M	This work
WBDacB	WB600/pBE-P*_amyQ_*-RBS0-SP*_dacB_*-*amyS*M	This work
WBNprE	WB600/pBE-P*_amyQ_*-RBS0-SP*_nprE_*-*amyS*M	This work
WBWapA	WB600/pBE-P*_amyQ_*-RBS0-SP*_wapA_*-*amyS*M	This work
WBVpr	WB600/pBE-P*_amyQ_*-RBS0-SP*_vpr_*-*amyS*M	This work
WBYwmD	WB600/pBE-P*_amyQ_*-RBS0-SP*_ywmD_*-*amyS*M	This work
WBBslB	WB600/pBE-P*_amyQ_*-RBS0-SP*_bslB_*-*amyS*M	This work
WBLytF	WB600/pBE-P*_amyQ_*-RBS0-SP*_lytF_*-*amyS*M	This work
WBPtufA	WB600/pBE-P*_tufA_*-RBS0-SP*_ykwD_*-*amyS*M	This work
WBPgsiB	WB600/pBE-P*_gsiB_*-RBS0-SP*_ykwD_*-*amyS*M	This work
WBPaprE	WB600/pBE-P*_aprE_*-RBS0-SP*_ykwD_*-*amyS*M	This work
WBPnprE	WB600/pBE-P*_nprE_*-RBS0-SP*_ykwD_*-*amyS*M	This work
WBPgapA	WB600/pBE-P*_gapA_*-RBS0-SP*_ykwD_*-*amyS*M	This work
WBPsodA	WB600/pBE-P*_sodA_*-RBS0-SP*_ykwD_*-*amyS*M	This work
WBRBS1	WB600/pBE-P*_gsiB_*-RBS1-SP*_ykwD_*-*amyS*M	This work
WBRBS2	WB600/pBE-P*_gsiB_*-RBS2-SP*_ykwD_*-*amyS*M	This work
WBRBS3	WB600/pBE-P*_gsiB_*-RBS3-SP*_ykwD_*-*amyS*M	This work
WBRBS4	WB600/pBE-P*_gsiB_*-RBS4-SP*_ykwD_*-*amyS*M	This work
WBRBS5	WB600/pBE-P*_gsiB_*-RBS5-SP*_ykwD_*-*amyS*M	This work
WBRBS6	WB600/pBE-P*_gsiB_*-RBS6-SP*_ykwD_*-*amyS*M	This work
WBRBS7	WB600/pBE-P*_gsiB_*-RBS7-SP*_ykwD_*-*amyS*M	This work
**Plasmids**		
pET-22b	Amp^r^, T7 promoter,SP*_pelB_*	Our laboratory
pBE-S	Amp^r^ (*E. coli*), Kan^r^ (*B. subtilis*), P_aprE_, SP*_aprE_*	Takara
pBE0	The derivative of the pBE-S plasmid pBE-P*_amyQ_*-RBS0-SP*_aprE_*	Our laboratory
pBE	The derivative of the pBE0 plasmid pBE-Px*-RBSx*-SPx*-*amyS*M	This work

* ‘x’ represents the serial number of different elements.

### Plasmid Construction

The *amyS* gene fragment was amplified from plasmid pET-28a-*amyS* (Hu et al., 2023), harboring the *amyS* gene from *G. stearothermophilus*, using primers amyS1-F/amyS1-R. Then, the fragment was ligated into the pBE0 (pBE-P*_amyQ_*-RBS0-SP*_aprE_*) plasmid through T4 DNA ligase at the restriction site with *Hin*dIII and *Sal*I. The ligation product pBE-P*_amyQ_*-RBS0-SP*_aprE_*-*amyS* was transformed into *E. coli* DH5α for the next manipulation. The randomly mutated *amyS* gene variant amyS* was amplified from plasmid pBE-P*_amyQ_*-RBS0-SP*_aprE_*-*amyS* using primers amyS2-F/amyS2-R, with the reaction system supplemented with 2 mM Mn^2+^ to introduce nucleotide sequence variations. Then, the fragment was ligated into the pET-22b plasmid through T4 DNA ligase at the restriction site with *Hin*dIII and *Sal*I, generating plasmid pET-22b-amyS*.

The fragment of triple mutant *amyS*M (T151A/K178E/T458A) was amplified from plasmid pET-22b-*amyS*M using primers *amyS*M-F/*amyS*M-R. Then, the fragment was ligated into the pBE0 plasmid through T4 DNA ligase at the restriction site with *Hin*dIII and *Sal*I, generating plasmid pBE-P*_amyQ_*-RBS0-SP*_aprE_*-*amyS*M.

The signal peptide fragment SP*_ydjM_* was amplified from plasmid pBE-S-SP*_ydjM_*-*amyS* using primers YdjM-F/YdjM-R. The fragment was ligated with the pBE-P*_amyQ_*-RBS0-SP*_aprE_*-*amyS*M using T4 DNA ligase at the restriction site with *Hin*dIII and *Sal*I to generate plasmids pBE-P*_amyQ_*-RBS0-SP*_ydjM_*-*amyS*M. The series of plasmids pBE-P*_amyQ_*-RBS0-SP_X_-*amyS*M (SP_X_ represents SP*_yqxL_*, SP*_abnAw_*, SP*_ykwD_*, SP*_dacB_*, SP*_nprE_*, SP*_vpr_*, SP*_wapA_*, SP*_ywmD_*, SP*_bslB_*, and SP*_lytF_*, respectively) with distinct signal peptides were obtained using the same method.

The promoter fragment P*_tufA_* (Meng et al., 2018) was amplified with the primers tufA-F/tufA-R using the *B. subtilis* 168 genomic DNA as template, and was linked with plasmid pBE-P*_amyQ_*-RBS0-SP*_ykwD_*-*amyS*M, digesting with *Kpn*I and *Xho*I, to generate plasmid pBE-P*_tufA_*-RBS0-SP*_ykwD_*-*amyS*M. The other plasmids pBE-P_X_-RBS0-SP*_ykwD_*-*amyS*M (P_X_ represents P*_gsiB_* (Zhang et al., 2017), P*_aprE_* (Zhang et al., 2017), P*_nprE_* (Zhang et al., 2017), P*_gapA_* (Meng et al., 2018) and P*_sodA_* (Meng et al., 2018), respectively) with different promoters were constructed by the same method.

The linearized pBE-P*_gsiB_*-RBS0-SP*_ykwD_*-*amyS*M fragment was generated by *Dpn*I digestion (37°C, 1 h) of the PCR product amplified from plasmid pBE-P*_gsiB_*-RBS0-SP*_ykwD_*-*amyS*M using primers RBS1-F/RBS1-R. Then, the linearized fragment was purified through gel extraction and subsequently subjected to the treatment of T5 exonuclease. In brief, 2 μL purified linearized fragment was mixed with 1 μL of T5 exonuclease, 4 μL 10 × NEBuffer 4 and 6 μL double-distilled water (ddH_2_O), and then incubated on ice for 5–10 minutes. The reaction mixture was transformed into *E. coli* DH5α to generate recombinant plasmid pBE-P*_gsiB_*-RBS1-SP*_ykwD_*-*amyS*M. The other plasmids pBE-P*_gsiB_*-RBSx-SP*_ykwD_*-*amyS*M (RBSx represents RBS2, RBS3, RBS4, RBS5, RBS6 and RBS7, respectively) with different RBS sequences were constructed using the same method.

### Screening in 96-well Plates

The recombinant plasmid pET-22b-amyS* was transformed into *E. coli* BL21. Monoclonal colonies expressing AmyS* on LB agar plates were inoculated into 96-well plates containing 400 μL of LB medium per well and incubated at 37°C and 220 rpm for 4 h. Then, the α-amylase activity in the supernatant was determined after induction with 0.5 mM IPTG (final concentration) and incubation at 18°C for 24 h. The fragment lacking the original SP sequence was amplified from pBE-P*_amyQ_*-SP*_aprE_*_-_*amyS*M using primers sp-F/sp-R. Then, the purified pBE-P*_amyQ_*-_-_*amyS*M fragment was linked with 173 different *B. subtilis* signal peptides (obtained from Secretory Protein Expression System, Takara, Japan) using the One Step Cloning Kit (Vazyme, China) to generate plasmids with different signal peptides pBE-P*_amyQ_*-SPs-*amyS*M. The series of plasmids was transformed into *E. coli* DH5α for plasmid propagation, followed by electroporation into *B. subtilis* 168. The recombinant strains (*B. subtilis* 168/pBE-P*_amyQ_*-SPs-*amyS*M) were inoculated in 96-well plates containing 400 μL LB medium, with *B. subtilis* 168/pBE-P*_amyQ_*-SP*_aprE_*_-_*amyS*M serving as a control. The plates were incubated at 37°C, 250 rpm for 10 h. The cultures were subsequently transferred to 96-deep-well plates containing 400 μL fresh LB medium and fermented under identical conditions for 48 h. Finally, the α-amylase activity in the supernatant was determined. The culture medium of *B. subtilis* was supplemented with 60 μg/mL kanamycin.

### Culture and Fermentation Conditions

#### Fermentation in shake flask

Fifty microliters of glycerol-stored bacteria, maintained at -80°C, were transferred to 5 mL LB medium and activated at 37°C and 200 rpm for 12 h. Subsequently, 2% (v/v) of this culture was transferred into 50 mL TB medium in a 250 mL shake flask. The α-amylase activity was assayed in the supernatant after 48 h of fermentation under identical conditions.

#### Fermentation in 5-L fermenter

Fifty microliters of glycerol-stored bacteria, maintained at -80°C, were activated in 20 mL LB medium at 37°C and 200 rpm for 12 h. Subsequently, the culture was transferred into 100 mL TB medium in a 500 mL shake flask (1%, v/v) and cultivated for 24 h under identical conditions. The seed culture was then inoculated into 5-L fermenters containing 2 L of TB medium (5%, v/v). The dissolved oxygen (DO) tension was maintained at 30% by automatically adjusting the stirrer speed (200–800 rpm). The pH was maintained at 7.0 by the automatic addition of NH_4_OH and H_3_PO_4_. The fermentation temperature was automatically controlled at 32°C. Antifoam was supplemented at 0.3% (v/v) as required. Upon DO reaching the upper trigger point of 60 %, continuous feeding was automatically started with a 5–10 mL/h feed rate to sustain metabolic activity. Kanamycin (60 μg/mL) was supplemented throughout *B. subtilis* cultivation. Culture samples were collected every 4 h for growth monitoring.

### Analytical Methods

#### Determination and analysis of AmyS activity

The activity of AmyS in the supernatant was determined using the modified dinitrosalicylic acid (DNS) assay method according to Hu et al. (Hu et al., 2023). The standard assay mixtures were assayed at 70°C for 5 min in 0.3 mL of 0.1 M citrate-phosphate buffer (pH 5.0 100 μL) containing 1.5% soluble starch (w/v, 100 μL) as substrate with 0.1 mL of diluted enzyme. The reaction was stopped by adding 0.3 mL DNS solution, and the mixture was incubated in a boiling water bath for 5 min. After cooling to room temperature, the absorbance of the supernatant at 540 nm was measured. The substrate and enzyme blanks were prepared in the same way as the analyzed sample, except that 0.1 mL of deionized water was added to the substrate (enzyme) solution instead of the enzyme solution. The A_540_ values for the substrate and enzyme blanks were subtracted from the A_540_ value for the analyzed sample. One unit of AmyS activity was defined as the amount of enzyme needed to release 1 μmol of reducing sugars per minute under standard assay conditions described above.

#### Starch plate assay

The AmyS activity in the supernatant was determined using a plate experiment in which an aliquot (2 μL) of the supernatant was inoculated into LB agar (1%) plates containing 2 % soluble starch. Then, the plates were incubated at 60°C for 1 h. The AmyS activities were evaluated by measuring the diameters of clear circles after staining with Lugol’s iodine solution. The strains with clear circles larger than those of the control strain exhibited higher enzymatic activity in the fermentation samples (Li et al., 2022b).

#### Sodium dodecyl sulfate-polyacrylamide gel electrophoresis (SDS-PAGE)

The fermentation broth was centrifuged at 8 000 × g for 20 min at 4°C to obtain the culture supernatants. 20 μL of supernatant was mixed with 5 μL of 5 × SDS-PAGE loading buffer, followed by heating at 100°C for 10 min. Subsequently, 8 μL of the denatured mixture was electrophoresed. The subunit molecular weight of recombinant enzymes was determined under denaturing conditions using 10% separating gels. Protein bands were visualized by staining with Coomassie Brilliant Blue R-250 according to standard protocols.

#### Biomass

During fermentation, the fermentation broth was diluted with 0.9% (w/v) NaCl solution. The optical density at 600 nm wavelength (OD_600_) was measured using a spectrophotometer (Shanghai Metash Instruments Co., Ltd) to assess cellular growth.

### Structural Analysis of AmyS

Structure Modeling. The wild-type GsAMY crystal structure (PDB ID: 4UZU) served as the template for modeling. The T151A/K178E/T458A triple mutant structure was generated using the SWISS-MODEL server (Guex and Peitsch, 1997). All structural visualizations were performed with PyMOL (Schrödinger, LLC). Molecular Docking. The substrate maltoheptaose (G7) was constructed in mol2 format via the Glycam Carbohydrate Builder. The protein model was preprocessed by adding hydrogen atoms and assigning protonation states using the LePro module. A docking box encompassing the active-site cleft (subsites -1 to -7) was defined. Molecular docking was executed with LeDock (Wang et al., 2016), generating multiple binding poses ranked by predicted binding free energy. Poses consistent with canonical GH13 family substrate-binding modes were manually selected for analysis.

### Molecular Dynamics Simulations of AmySM-maltoheptaose Complex

Molecular dynamics (MD) simulations were conducted for the wild-type (WT) and M3 (T151A/K178E/T458A) AmySM-maltoheptaose complexes. The enzyme structure and the carbohydrate ligand were described using the Amber ff14SB (Maier et al., 2015) and GLYCAM06j (Kirschner et al., 2008) force fields, respectively. Each complex was solvated in an explicit TIP3P (Jorgensen et al., 1983) water box with a minimum distance of 16 Å between the solute and the box boundary, and the system was neutralized by adding Na⁺ and Cl⁻ ions. All systems were subjected to energy minimization followed by equilibration prior to production runs. Heating was conducted from 0 to 300 K under the NVT ensemble with positional restraints on heavy atoms, followed by equilibration under constant pressure (1 atm) with gradual release of restraints. Production simulations of 100 ns were performed in the NPT ensemble at 300 K and 1 atm using Langevin dynamics for temperature coupling and a Berendsen barostat for pressure control. Long-range electrostatics were treated using the particle mesh Ewald (PME) (Essmann et al., 1995) method with a nonbonded cutoff of 12 Å. All bonds involving hydrogen atoms were constrained using the SHAKE (Kräutler et al., 2001) algorithm, allowing a 2 fs integration timestep.

Trajectory analyses were performed using cpptraj to compute backbone root mean square deviation (RMSD), per-residue root mean square fluctuation (RMSF), and linear interaction energy (LIE) between the enzyme and the substrate. The LIE values were averaged over equilibrated trajectories to evaluate the relative binding affinity between WT and M3. All simulations were carried out using the GPU-accelerated AMBER 24 (Rao et al., 2022) package.

### Statistical Analysis

All of the experiments mentioned above were performed independently in triplicate. One-way analysis of variance (ANOVA) was used for statistical analysis. Data charts were conducted using GraphPad Prism software, with results represented as average values with standard deviations.

## Results and Discussion

### Recombinant Expression of *amyS* in *B. Subtilis* WB600

**Fig. 1.**
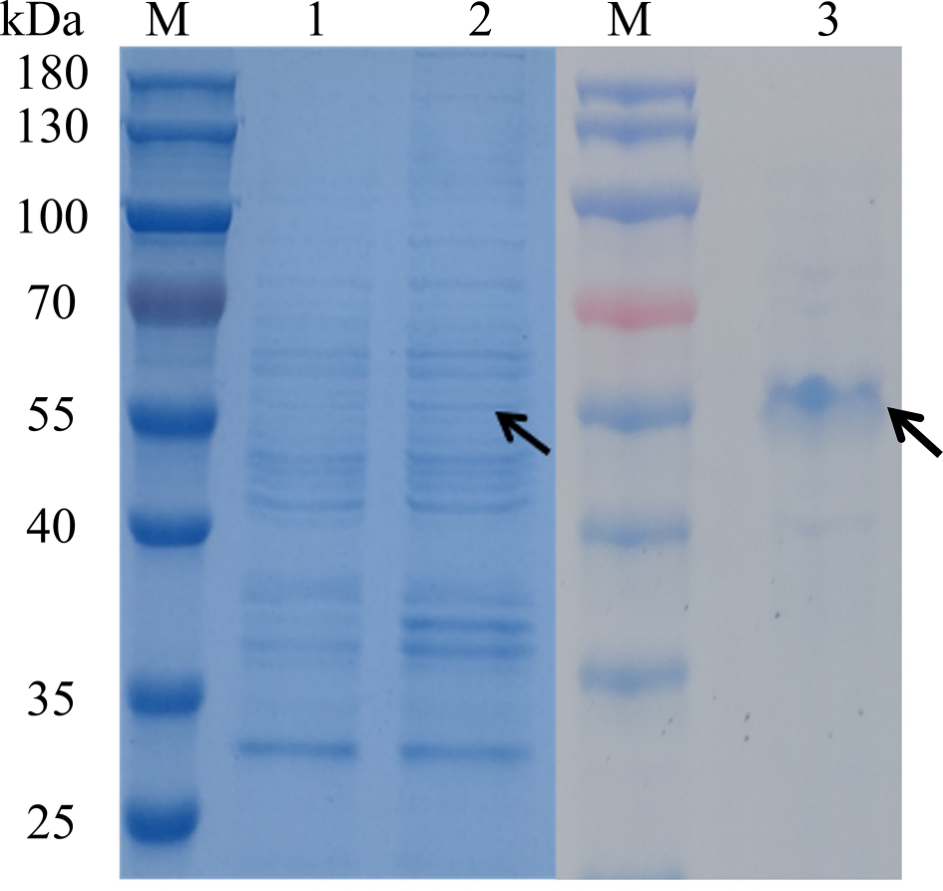
SDS-PAGE analysis of recombinant AmyS expression in *B. subtilis* WB600. Lane M, protein ladder; Lane 1, extracellular protein profile of control strain WBK (WB600/pBE) supernatant; Lane 2, extracellular protein profile of experimental strain WBSW (WB600/pBE-*amyS*) supernatant; Lane 3, purification of AmyS protein from the extracellular supernatant of experimental strain WBSW. The AmyS band is indicated by a black arrowhead.

The *amyS* from *G. stearothermophilus* was successfully expressed and secreted in *B. subtilis* WB600, as confirmed via shake-flask fermentation. The recombinant strain WBSW exhibited an extracellular α-amylase activity of 44.36 ± 2.02 U/mL. SDS-PAGE analysis (Fig. 1, Lane 1) showed a distinct band at approximately 55 kDa, consistent with the predicted molecular weight of AmyS. These results confirm the successful expression and extracellular secretion of recombinant AmyS in *B. subtilis*.

### High-throughput Screening of AmyS Mutants With Enhanced α-amylase Activity

**Fig. 2.**
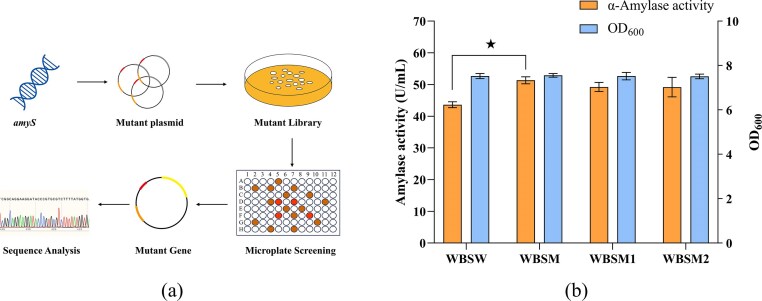
Screening for AmyS mutants with enhanced α-amylase activity. (a) Flowchart for screening activity-enhanced AmyS mutants of α-amylase. (b) The extracellular activities and cell densities (OD_600_) of different AmyS mutants in shake-flask fermentation. WBSM, (WB600/pBE-amyS*, recombinant harboring triple mutant T151A/K178E/T458A); WBSM1, (WB600/pBE-amyS*, recombinant harboring mutant T451M); WBSM2, (WB600/pBE-amyS*, recombinant harboring mutant Q480P). Error bars represent the standard deviation from three independent experimental replicates. ‘★’ indicates that AmySM activity was significantly increased compared to the control (*P* < 0.05).

To enhance catalytic activity, a mutant library of 1 500 variants was constructed via error-prone PCR-mediated random mutagenesis of the *amyS* gene (Fig. 2a). Of the mutants generated, three variants (T151A/K178E/T458A, T451M, and Q480P) showed the greatest potential for enhanced activity. Shake-flask fermentation of the recombinant *B. subtilis* WB600 strains revealed significantly enhanced extracellular α-amylase activities: the control strain (WBSW) reached 43.63 ± 0.93 U/mL, while the variant-expressing strains WBSM, WBSM1, and WBSM2 produced 51.34 ± 1.11 U/mL, 49.21 ± 1.43 U/mL, and 49.17 ± 3.08 U/mL, respectively. Particularly, the WBSM strain, expressing the T151A/K178E/T458A triple mutant, exhibited a 17.67% increase in enzymatic activity relative to the control strain WBSW. Furthermore, the AmySM mutant (T151A/K178E/T458A) maintained the optimal temperature and pH profile of wild type enzyme while achieving a 6.37% improvement in specific activity (Figure S1a-c).

To investigate the structural underpinnings of the enhanced specific activity in the AmySM triple mutant (T151A/K178E/T458A), we conducted an in-depth analysis of the mutation sites and evaluated their influence on enzyme conformation and substrate binding affinity. As illustrated in Figure S2a, the AmySM structure consists of three domains: a catalytic core (green), a B-domain (purple), and a C-domain (cyan). The T151A and K178E mutations are located within the B-domain, whereas T458A resides in the C-domain. Notably, K178 is positioned in the closest proximity to the catalytic center, implying its potential regulatory role. In the substrate-bound model (Figure S2b), three aromatic residues (Y200, W167, and W140) within the B-domain engage in π-π stacking interactions with maltoheptaose at subsites -4 to -6, highlighting the critical role of this region in substrate binding. In the wild-type GsAMY, residue K178 engages in a salt bridge with E129, contributing to the structural stabilization of local β-sheet (Figure S2c). The K178E mutation abolishes this salt bridge, resulting in a conformational shift from a β-sheet to a loop structure and enhancing local flexibility (Figure S2d). The enhanced flexibility of the B-domain may promote substrate accommodation, a phenomenon consistent with earlier reports suggesting that increased local flexibility near the active site improves substrate binding and catalytic turnover in glycoside hydrolases.

To gain deeper insights into the conformational dynamics and stability, we conducted 100 ns molecular dynamics (MD) simulations on both the WT and the M3 mutant of AmySM in complex with maltoheptaose. Consistent with the initial structural observations, the MD trajectories indicated that the M3 mutant exhibited a modest increase in overall flexibility, as evidenced by slightly higher backbone root mean square deviation (RMSD) values than the WT (Figure S3a-b). Specifically, the B domain surrounding the mutation sites (Figure S3c) showed markedly higher root mean square fluctuation (RMSF) values (Figure S3d), confirming that the K178E substitution introduces greater mobility, in full agreement with our structural analysis.

In the M3 mutant, the enhanced flexibility of this loop repositions the nearby aromatic residues W140 and W167 toward the maltoheptaose (Figure S3d). These enhanced hydrophobic π-stacking interactions strengthen the contact between the B domain and the substrate, thereby providing a structural rationale for the improved binding. This interpretation is directly supported by linear interaction energy (LIE) analysis, which showed a decrease in the binding free energy from –222 ± 17 kcal/mol for the WT to –251 ± 30 kcal/mol for the M3 mutant, confirming that the optimized substrate–enzyme interactions underlie the increased catalytic efficiency. Our findings collectively underscore that fine-tuning structural flexibility is a critical mechanism for regulating enzymatic function, thereby validating directed evolution as a powerful strategy for enhancing the catalytic activity (Bessler et al., 2009; Wong et al., 2004).

### Screening of Signal Peptides to Enhance AmySM Enzyme Activity

**Fig. 3.**
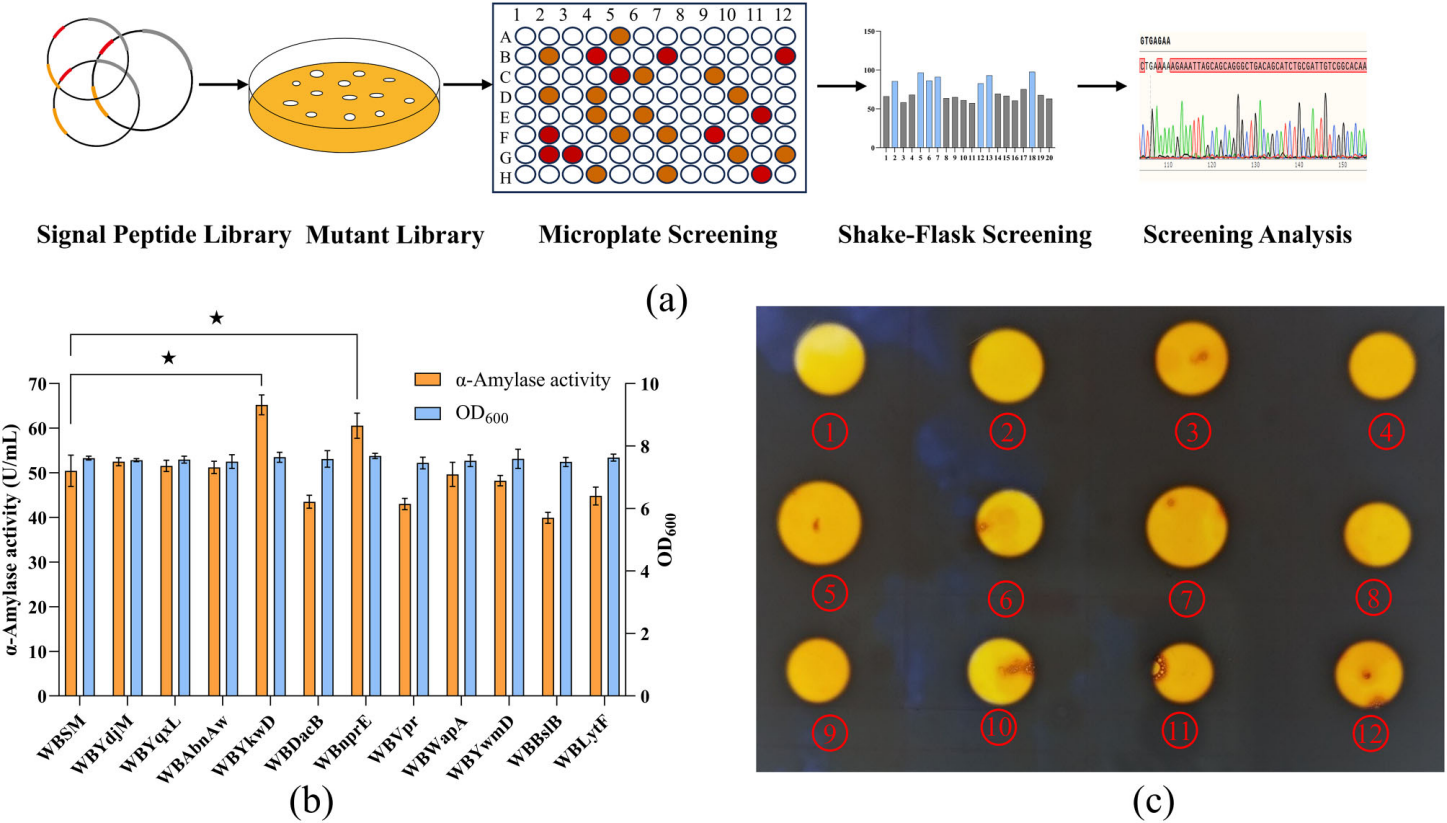
Optimization of signal peptide. (a) Flowchart for screening of signal peptides. (b) Evaluation of recombinants with different signal peptides for α-amylase activity and cell growth in shake-flask fermentation. (c) Starch plate assay for α-amylase activity verification in extracellular fermentation supernatants of recombinant strains harboring distinct signal peptides. ①-⑫ represent halo zones corresponding to fermentation supernatants from recombinants WBSM, WBYdjM, WBYqxL, WBAbnAw, WBYkwD, WBDacB, WBNprE, WBVpr, WBWapA, WBYwmD, WBBslB, and WBLytF, respectively. Error bars represent the standard deviation from three independent experimental replicates. ‘★’ indicates that AmySM activity was significantly increased compared to the control (*P* < 0.05).

To improve extracellular secretion, we developed a high-throughput signal peptide screening platform based on a commercial library (Fig. 3a). Screening of 1 000 *B. subtilis* 168-derived signal peptides led to the initial identification of 11 candidates capable of enhancing AmySM secretion (Figure S4, Table S2). Subsequently, we constructed recombinant *B. subtilis* WB600 strains, WBYdjM, WBYqxL, WBAbnAw, WBYkwD, WBDacB, WBNprE, WBVpr, WBWapA, WBYwmD, WBBslB and WBLytF, each carrying one of the candidate signal peptides, and evaluated their extracellular α-amylase production. Following 48 h of shake-flask fermentation, α-amylase activities were measured for both the control strain (harboring SP*_aprE_*) and the 11 recombinant strains. The corresponding activities were 50.44 ± 3.51, 52.47 ± 0.88, 51.55 ± 1.28, 51.22 ± 1.38, 65.23 ± 2.33, 43.51 ± 1.45, 60.55 ± 2.81, 43.02 ± 1.26, 49.66 ± 2.72, 48.23 ± 1.17, 39.92 ± 1.25, and 44.81 ± 2.0 U/mL, respectively (Fig. 3b). Among the engineered strains, WBYkwD and WBNprE demonstrated the highest α-amylase activities, with increase of 29.32% and 20.04%, respectively, compared to the control strain WBSW. Moreover, the OD_600_ values of all strains showed no significant differences, ruling out the possibility that variations in expression levels resulted from disparities in cellular growth. This confirms that the increased production of AmySM was directly attributable to the efficiency of signal peptide-mediated secretion. These results were further corroborated by starch plate assays, in which the fermentation supernatants of strains WBYkwD and WBNprE formed noticeably larger hydrolysis halos than that of the control (Fig. 3c), aligning with the observed enzymatic activity datas.

This study systematically evaluated the secretory efficiency of 11 signal peptides for α-amylase in *B. subtilis*, revealing SP*_ykwD_* as the most effective for enhancing α-amylase activity. This observation is consistent with reports by Liu et al., in which efficient extracellular secretion of PsDex1711 was mediated by SP*_ykwD_*, a signal peptide native to the *ykwD* gene. The YkwD protein, an SCP-domain-containing calcium-chelating serine protease, depends on its cognate signal peptide SP*_ykwD_* for proper secretion and interaction with the spore cortex (Keijser et al., 2007; Liu et al., 2024). Based on these findings, we propose that the SP*_ykwD_* may possess intrinsic properties that facilitate highly efficient recombinant proteins secretion in *B. subtilis*. However, to the best of our knowledge, no previous studies have reported the use of SP*_ykwD_* for enhancing α-amylase secretion in this host. This gap may stem from the structural and biochemical diversity among α-amylases from different sources, underscoring the need for enzyme-specific signal peptide selection to achieve optimal secretion (Chen et al., 2014; Li et al., 2022b; Yao et al., 2019). Moreover, the signal peptide SP*_nprE_*, known for mediating the secretion of neutral protease NprE, which constitutes over 70% of the total extracellular protease activity in *B. subtili* (Yang et al., 1984), also demonstrated high efficacy in facilitating α-amylase secretion. Therefore, SP*_nprE_* appears to harbor a similarly strong inherent secretion capability. In fact, it is well-established as a high-performance signal peptide for the secretion of heterologous protein in*B. subtilis* (Fu et al., 2018). In a comparative study of seven signal peptides (SP*_nprE_*, SP*_aprE_*, SP*_wapA_*, SP*_yncM_*, SP*_amyE_*, and SP*_sacB_*) for α-amylase secretion in *B. subtilis*, Chen et al. found that SP*_nprE_* conferred the highest extracellular activity (260 U/mL), exceeding other candidates by 1.44- to 3.38-fold (Chen et al., 2014). In *B. subtilis*, the translocation of proteins from the cytoplasm to the extracellular environment is mediated by a multi-component secretion machinery, in which each element performs specialized functions such as transport, translocation, cleavage, and release of the mature protein. Efficient secretion critically relies on the tightly orchestrated coordination among these components (Fu et al., 2018; Yao et al., 2019). However, the underlying mechanisms responsible for high secretion efficiency mediated by SP*_ykwD_* and SP*_nprE_* remain to be elucidated.

### Promoter Optimization for Enhancing AmySM Enzyme Activity

**Fig. 4.**
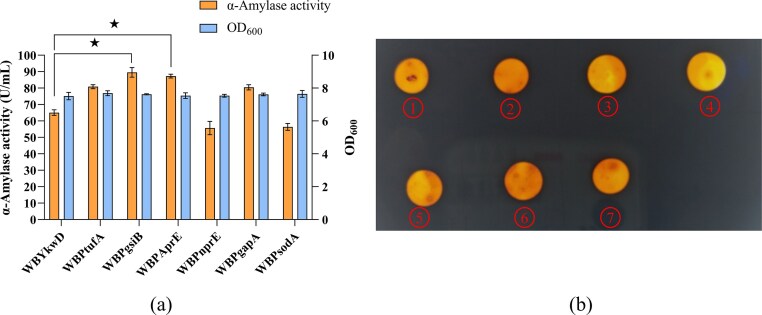
Optimized Promoter. (a) Evaluation of extracellular α-amylase activities and cell densities of strains with different promoters in shake-flask fermentation. (b) Starch plate assay for α-amylase activity verification in extracellular supernatants of recombinant strains driving by different promoters. ①-⑦ represent halo zones corresponding to fermentation supernatants from recombinants WBYkwD, WBPtufA, WBPgsiB, WBPaprE, WBPnprE, WBPgapA and WBPsodA, respectively. Error bars represent the standard deviation from three independent experimental replicates. ‘★’ indicates that AmySM activity was significantly increased compared to the control (*P* < 0.05).

To enhance *amyS*M expression in *B. subtilis* WB600, we performed systematic promoter screening by replacing the native P*_amyQ_* in the base plasmid pBPamyQ (pBE-P*_amyQ_*-RBS0-SP*_ykwD_*-*amyS*M) with six strong promoters: P*_tufA_*, P*_gsiB_*, P*_aprE_*, P*_nprE_*, P*_gapA_*, and P*_sodA_*. The resulting recombinant plasmids were transformed into *B. subtilis* WB600 for expression evaluation. The control strain WBYkwD, which retained original promoter P*_amyQ_*, exhibited an extracellular α-amylase activity of 65.07 ± 1.72 U/mL. The recombinant strains WBPtufA, WBPgsiB, WBPaprE, WBPnprE, WBPgapA, and WBPsodA exhibited extracellular α-amylase activities of 80.84 ± 1.21, 89.54 ± 2.95, 87.30 ± 1.17, 55.69 ± 4.06, 80.48 ± 1.57, and 56.40 ± 2.09 U/mL, respectively. Among these, strains WBPgsiB and WBPaprE showed the most notable improvements, with activity increases of 37.61% and 34.16%, respectively, compared to WBYkwD. Furthermore, all strains exhibited comparable growth profiles, with cell density ranging from 7.51 ± 0.23 to 7.69 ± 0.15, indicating that the variations in enzymatic activity were not due to differences in biomass but driven by promoter strength. Consistent with the enzymatic activity data, starch plate assays showed that the fermentation supernatants of strain WBPgsiB and WBPaprE formed markedly larger hydrolysis zones than that of the control WBYkwD (Fig. 4b), further validating the efficacy of promoter screening.

In this study, promoters P*_gsiB_* and P*_aprE_* significantly enhanced the recombinant expression of AmySM in *B. subtilis* WB600. Notably, P*_gsiB_* is characterized as a strong auto-inducible promoter activated under environmental stresses and energy limitation, regulating downstream gene transcription via the Sigma-B factor (Hecker et al., 2003). For instance, Nguyen et al. demonstrated that P*_gsiB_* effectively drives recombinant β-galactosidase expression in *B. subtilis*, with significantly elevated yields under stress conditions such as acid shock, ethanol exposure, or heat shock (Nguyen et al., 2005). Moreover, when coupled with a strong RBS downstream, P*_gsiB_* further enhances mRNA stability and translational efficiency (Jürgen et al., 1998). Similarly, the promoter P*_aprE_* displays strong transcriptional activity in *B. subtilis* and has been successfully employed for the efficient extracellular expression of several heterologous proteins, such as BLAase (Niu et al., 2022), α-amylase (Chen et al., 2014) and keratinase (kerBv) (Gong et al., 2020). Its performance is further enhanced by downstream UTR elements that contribute to mRNA stabilization and improved expression (Chen et al., 2014; Hambraeus et al., 2002).

Although P*_gsiB_* and P*_aprE_* significantly enhanced AmySM expression in *B. subtilis* in this study, their performance appears to be protein-dependent, as previous studies have reported limited efficacy of P*_gsiB_* and P*_aprE_* for nattokinase (Liu et al., 2019b) and staphylokinase (Kim et al., 2008), respectively. These observations indicate that promoter characteristics, including origin, adjacency to the target gene, and features of the 5’-untranslated region, are key determinants of transcriptional strength (Liu et al., 2019b). Thus, the selection of an optimal promoter must account for target gene-specific features to ensure high-level expression.

### Regulation of RBS for Enhancing AmySM Enzyme Activity

**Fig. 5.**
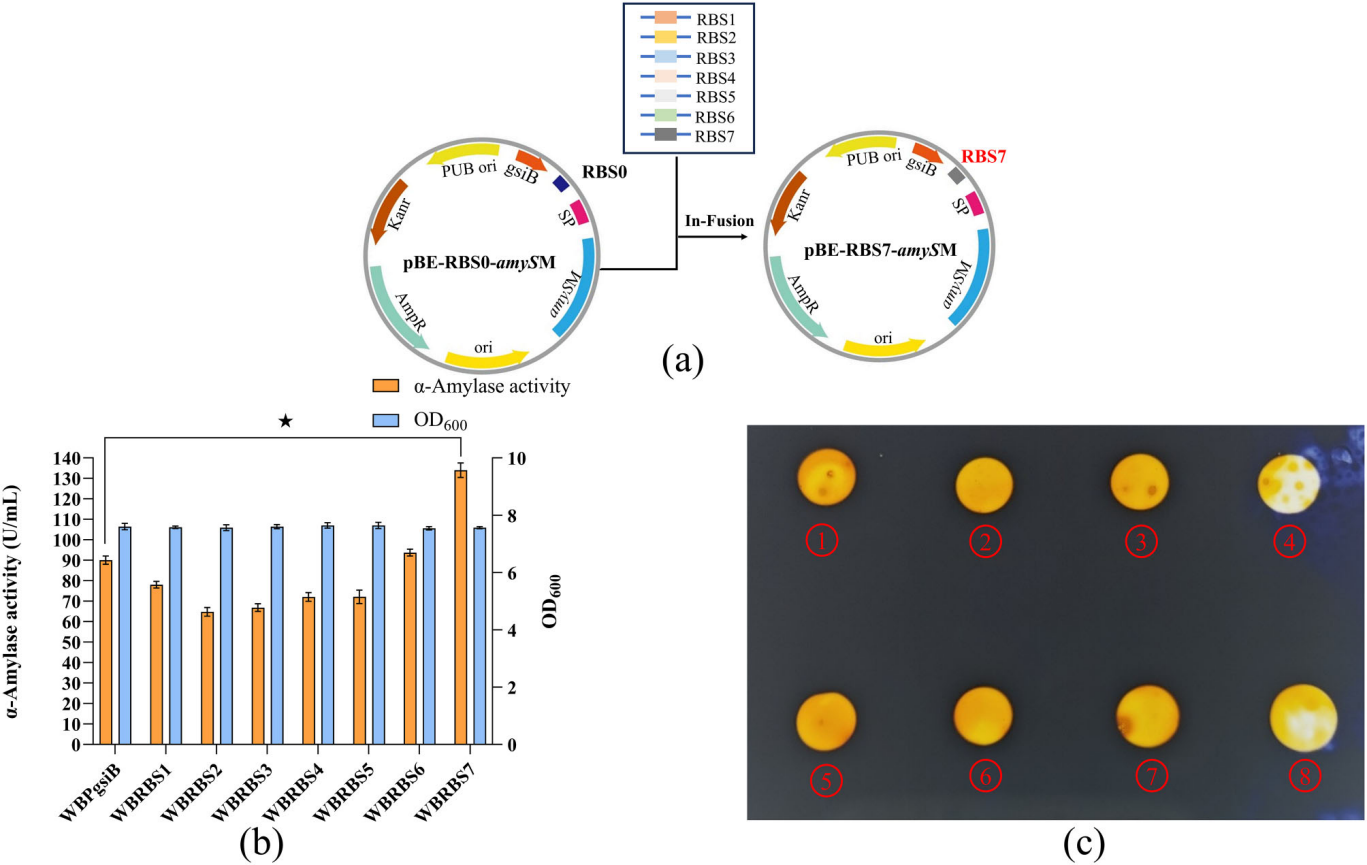
Optimized RBS Sequence. (a) Schematic diagram of RBS0 sequence replacement on plasmid pBRBS0. (b) Evaluation of extracellular α-amylase activities and cell densities of strains with different RBS sequences in shake-flask fermentation. (c) Starch plate assay for α-amylase activity verification in extracellular supernatants of recombinant strains harboring different RBS sequences. ①-⑧ represent halo zones corresponding to fermentation supernatants from recombinants WBPgsiB, WBRBS1, WBRBS2, WBRBS3, WBRBS4, WBRBS5, WBRBS6 and WBRBS7, respectively. Error bars represent the standard deviation from three independent experimental replicates. ‘★’ indicates that AmySM activity was significantly increased compared to the control (*P* < 0.05).

To further increase AmySM production in *B. subtilis* WB600, the RBS sequences, a key sequence governing gene translation initiation through recruitment and binding of ribosome to the transcribed mRNA, were optimized by replacing the original RBS0 in the plasmid pBRBS0 (pBE-P*_gsiB_*-RBS0-SP*_ykwD_*-*amyS*M) with seven rationally designed variants (RBS1–RBS7; Table S3) as illustrated in Fig. 5a. Shake-flask fermentation was conducted to assess the extracellular enzymatic activities and cell densities of engineered strains *B. subtilis* strains harboring different RBS sequences. The recombinant strains WBPgsiB, WBRBS1, WBRBS2, WBRBS3, WBRBS4, WBRBS5, WBRBS6 and WBRBS7 exhibiting amylase activities of 90.05 ± 2.04, 77.99 ± 1.61, 64.79 ± 2.10, 66.86 ± 1.86, 72.06 ± 2.10, 72.15 ± 3.28, 93.71 ± 1.66 and 134.02 ± 3.54 U/mL, respectively (Fig. 5b). Notably, the recombinant strain WBRBS7 exhibited a 48.83% increase in α-amylase activity relative to the control strain WBPgsiB (pBE-P*_gsiB_*-RBS0-SP*_ykwD_*-*amyS*M), while the remaining variants resulted in reduced expression to varying degrees. These results demonstrated the substantial impact of RBS-mediated translational regulation on AmySM production. All recombinant strains showed comparable cell densities, with OD_600_ values ranging from 7.56 ± 0.08 to 7.76 ± 0.11, confirming that the enhanced enzymatic activity was not caused by altered growth but resulted directly from optimized translation efficiency. Furthermore, the sizes of hydrolysis zones formed by the recombinant strains carrying different RBS variants on starch plates correlated well with their enzymatic activity (Fig. 5c), providing additional visual validation of the RBS engineering effect.

In *B. subtilis*, the strength of RBS sequences exerts a direct influence on translation initiation efficiency, capable of modulating protein expression over a range of several orders of magnitude (Vellanoweth and Rabinowitz, 1992). Consistent with these findings, Mao et al. reported a 1.31-fold increase in diacetylchitobiose deacetylase activity using a similarly optimized RBS variant (RBS7) (Mao et al., 2021). This highlights the considerable potential of RBS sequence optimization for fine-tuning protein translation in *B. subtilis*. The enhancement of AmySM enzyme activity by RBS7 was consistent with expectations, whereas the other tested RBS sequences (RBS1-RBS6) showed no significant improvement. This limited success rate, further supported by the findings of Peng et al., underscores the context-dependent nature of RBS activity and suggests that the mechanistic principles governing their efficiency in heterologous expression systems are not yet fully generalizable (Peng et al., 2020). Compared to the other genetic regulatory components, the RBS offers practical advantages due to its compact size, ease of engineering, and minimal interference from complex cellular regulatory networks. Consequently, RBS optimization serves as a dual-purpose strategy that not only enhances recombinant gene expression efficiency but also redirects metabolic flux toward target protein synthesis. For instance, rational modulation of RBS strength in key metabolic pathway genes has been shown to result in a 2-fold difference in pulcherriminic acid production (Rao et al., 2024).

### Evaluation of Extracellular α-amylase Activities of Recombinant WBRBS7 in 5–L Fermenter

**Fig. 6.**
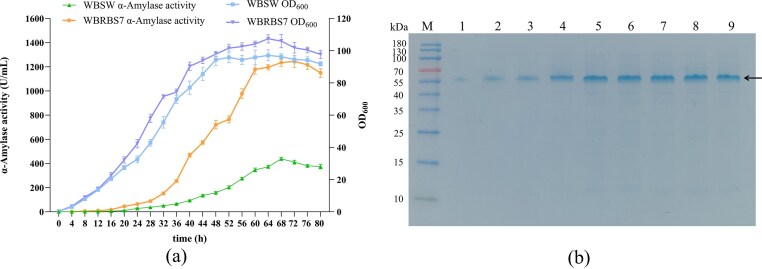
Verification of Recombinant Strains in a 5-L Fermenter. (a) The extracellular α-amylase activities and cell densities of the recombinant strain WBRBS7 cultured in 5–L fermenter. (b) SDS-PAGE analysis of purified AmySM expression in recombinant strain WBRBS7 cultivating for 16, 24, 32, 52, 60, 64, 68, 72, and 80 h in 5-L fermenter. Lane M, protein marker; lanes 1 to 9, AmySM expression in recombinant strain WBRBS7 cultivating for 16, 24, 32, 52, 60, 64, 68, 72, and 80 h in 5-L fermenter. The protein molecular weight of AmySM is 55 kDa, which is indicated by the black arrowhead. Error bars represent the standard deviation from three independent experimental replicates.

In initial shake-flask fermentation, the recombinant strain WBRBS7 showed a 3.02-fold increase in extracellular α-amylase activity compared to the parental strain WBSW. To further validate the cumulative effect of the multistage engineering strategy, WBRBS7 was cultivated in a 5-L bioreactor to evaluate its α-amylase production under controlled fermentation conditions. As shown in Fig. 6a, the recombinant strain WBRBS7 reached a maximum extracellular α-amylase activity of 1244.17 ± 48.66 U/mL after 72 h of bioreactor fermentation, a 2.84-fold increase over the initial strain WBSW (437.81 ± 12.67 U/mL at 68 h). Notably, this titer also represents a 9.28-fold enhancement compared to the performance of WBRBS7 under shake-flask fermentation. To further characterize the expression profile of AmySM in strain WBRBS7 during 5-L bioreactor fermentation, we performed SDS-PAGE analysis (Fig. 6b). The results indicated that extracellular AmySM accumulation reached its maximum at the late fermentation stage (72 h), confirming the efficient secretion of multi-optimized AmySM in *B. subtilis* WB600. While previous studies have primarily explored the structure-function relationships of *G. stearothermophilus* α-amylase (Hu et al., 2023; Kikani and Singh, 2021), we report the first high-cell-density recombinant expression of *G. stearothermophilus* α-amylase in *B. subtilis* using a 5-L fermenter, achieving a final OD_600_ of 105.94, a maximum α-amylase activity of 1244.17 ± 48.66 U/mL, and a specific activity of 585.60 ± 10.18 U/mg.

## Conclusion

Employing a combinatorial engineering approach that integrated directed evolution with systematic optimization of the signal peptide, promoter, and RBS, we significantly enhanced the extracellular yield of *Geobacillus stearothermophilus*, derived AmySM in *B. subtilis* WB600. The engineered strain WBRBS7 achieved the highest extracellular α-amylase activity of 134.02 ± 3.54 U/mL in shake-flask fermentation at 48 h. Moreover, When scaled up to a 5-L bioreactor, WBRBS7 reached a maximum AmySM activity of 1244.17 ± 48.66 U/mL, representing a 2.84-fold increase over the control strain WBSW. The strategies developed in this study establish a robust foundation and offer practical tools for the industrial-scale AmySM production. Future work will apply quantitative proteomic to uncover the molecular mechanisms responsible for enhanced AmySM expression and secretion.

## Supplementary Material

kuaf036_Supplemental_Files
