# Supplementary material for: Synergistic strategies for high production of Geobacillus stearothermophilus α-amylase in Bacillus subtilis
Source: J Ind Microbiol Biotechnol. 2025 Dec 19;53:kuaf036. doi: 10.1093/jimb/kuaf036 (PMC12784944; doi:10.1093/jimb/kuaf036)
Supplement: kuaf036_Supplemental_Files [file kuaf036_supplemental_files.zip › Revised Supplementary Materials.docx]

# **Supplementary materials**

**Title**: **Synergistic strategies for high production of *Geobacillus stearothermophilus* α-amylase in *Bacillus subtilis***

**Authors**: Deming Rao^a^, Changhui Wang^a^, Xiaolin Li^b^, Wei Shen^a^, Qiang Liu^a^, Zerui Li ^a^, Shiyu Pi ^a^, Zhenggang Han^a*^, Jiangke Yang^a*^

**Affilications**:

^a^ School of Life Science and Technology, Wuhan Polytechnic University, Wuhan 430023, People's Republic of China

^b^ Tianjin Institute for Drug Control (TIDC), 98 Guizhou Road, Tianjin, 300070, PR China.

*** Corresponding author**:

Zhenggang Han^a*^

School of Life Science and Technology, Wuhan Polytechnic University, Wuhan 430023, China.

E-mail: zhengganghan@whpu.edu.cn

Jiangke Yang^a*^

School of Life Science and Technology, Wuhan Polytechnic University, Wuhan 430023, China.

E-mail: yang.jiangke@whpu.edu.cn.

## **1. Primers**

## ‌**2. Characterization of enzymatic properties of AmySM**

### ‌**2.1 Protein purification**

AmySM was purified using nickel-NTA (Ni-NTA) affinity chromatography, as described in reference (Rao et al., 2023). The supernatant of WBSM (WB600/pBE-*amyS*M) culture broth was collected by centrifugation at 8,000×g for 20 min at 4 °C. The concentrated supernatant was loaded onto an Ni-NTA column. Initial washing was performed with Buffer A (50 mM phosphate-buffered saline (PBS), 100 mM imidazole, pH 6.5) to remove nonspecifically bound contaminants. Target protein AmySM was then eluted with Buffer B (50 mM PBS, 300 mM imidazole, pH 6.5), followed by dialysis against a stabilizing solution (0.2 M Na₂HPO₄/0.1 M citrate buffer, pH 6.0) at 4 °C. Purified fractions were aliquoted and cryopreserved at -20 °C. Notably, all purification buffers were pre-equilibrated to 4 °C to minimize protein degradation, and final AmySM concentrations were quantified via the Bradford assay.

### ‌**2.2 Optimal temperature and pH‌ for α-amylase activity determination**

To obtain the optimal temperature for α-amylase activity determination, the amylase-catalyzed reaction mixture was cultured in a pH 6.0 buffer with 1.5% soluble starch substrate under 30, 40, 50, 60, 70, 80, and 90 °C, respectively, for 5 min. The reaction was stopped by adding 0.3 mL DNS solution, and the mixture was incubated in a boiling water bath for 5 min. After cooling to room temperature, the absorbance of the supernatant at 540 nm was measured. Relative activity (%) was calculated with maximum activity set as 100% (Figure S1 a).

‌To obtain the optimal pH for α-amylase activity determination, the amylase-catalyzed reaction mixture was cultured in pH 3.0, 4.0, 5.0, 6.0, 7.0, and 8.0 buffers with 1.5% soluble starch substrate under 90 °C for 5 min. Relative activity (%) was calculated with maximum activity set as 100% (Figure S1 b).

The highest activity of AmyS and AmySM was obtained from cultures incubated at 70  °C and pH 5.0.

### ‌**2.3 Specific activity assay‌**

The specific activity of wild-type AmyS was defined as 100%. The specific activity of the mutant AmySM was expressed as a relative percentage (Figure S1 c).

## **3. Structural analysis of AmySM**

## ‌**4. Signal peptide screening**

## **5. RBS sequences**

## **6. References**

Rao, D., Huo, R., Yan, Z., Guo, Z., Liu, W., Lu, M., Luo, H., Tao, X., Yang, W., Su, L., Chen, S., Wang, L., Wu, J., (2023). Multiple approaches of loop region modification for thermostability improvement of 4,6-α-glucanotransferase from *Limosilactobacillus fermentum* NCC 3057. International Journal of Biological Macromolecules, 233, 123536. <https://doi:https://doi.org/10.1016/j.ijbiomac.2023.123536>.

**Table**

**Table S1.** Primers

| Primer | Sequence (5’-3’) | Restriction Enzymes |
| --- | --- | --- |
| amyS1-F | TCCGAATTC*AAGCTT*GCCGCACCGTTTAACC | *Hin*d Ⅲ |
| amyS1-R | CTAGACTGCAG*GTCGAC*TCTTTGAACATAAATTGAAACCGACC | *Sal* Ⅰ |
| amyS2-F | CTAGACTGCAG*GTCGAC*GCCGCACCGTTTAACC | *Sal* Ⅰ |
| amyS2-R | TCCGAATTC*AAGCTT*TCTTTGAACATAAATTGAAACCGACC | *Hin*d Ⅲ |
| amySM-F | TCCGAATTC*AAGCTT*GCCGCACCGTTTAACC | *Hin*d Ⅲ |
| amySM-R | CTAGACTGCAG*GTCGAC*TCTTTGAACATAAATTGAAACCGACC | *Sal* Ⅰ |
| pBE-F | GA*GTCGAC*CTGCAGTCTAGACATCACCATCATCACCACTA | *Sal* Ⅰ |
| pBE-R | GC*AAGCTT*GAATTCGGATCCGAGCTCCATATGTGC | *Hin*d Ⅲ |
| sp-F | G*CGGCCG*GTGCACATATGGAG | *Eag* Ⅰ |
| sp-R | CCTCTCTTAC*CTCGAG*ACGCG | *Xho* Ⅰ |
|  |  |  |
| YdjM-F | CC*CTCGAG*GTAAGAGAGGATGTTGAAGAAAGTCATTTTAGCC | *Xho* Ⅰ |
| YdjM-R | CAC*CGGCCG*CCGCACTGGCATCTGATG | *Eag* Ⅰ |
| YqxL-F | CC*CTCGAG*GTAAGAGAGGATGTTTAAGAAATTACTTTTAG | *Xho* Ⅰ |
| YqxL-R | CAC*CGGCCG*CAGCTTTGGCATGTCCATC | *Eag* Ⅰ |
| AbnAw-F | CC*CTCGAG*GTAAGAGAGGATGAAAAAGAAAAAAACATGG | *Xho* Ⅰ |
| AbnAw-R | CAC*CGGCCG*CTGCCTCTGCGGGAG | *Eag* Ⅰ |
| YkwD-F | CC*CTCGAG*GTAAGAGAGGATGAAGAAAGCATTTATTTTATCTGC | *Xho* Ⅰ |
| YkwD-R | CAC*CGGCCG*CCGCTGATGCTTGCTG | *Eag* Ⅰ |
| DacB-F | CC*CTCGAG*GTAAGAGAGGATGCGCATTTTCAAAAAAGC | *Xho* Ⅰ |
| DacB-R | CAC*CGGCCG*CAGCATGTGCTGTATTCAC | *Eag* Ⅰ |
| NprE-F | CC*CTCGAG*GTAAGAGAGGATGGGTTTAGGTAAGAAATTGTC | *Xho* Ⅰ |
| NprE-R | CAC*CGGCCG*CAGCCTGAACACCTGGC | *Eag* Ⅰ |
| WapA-F | CC*CTCGAG*GTAAGAGAGGATGAAAAAAAGAAAGAGGCGAAAC | *Xho* Ⅰ |
| WapA-R | CAC*CGGCCG*CTTTTGCTAGTACATCGGCTG | *Eag* Ⅰ |
| Vpr-F | CC*CTCGAG*GTAAGAGAGGATGAAAAAGGGGATCATTCGC | *Xho* Ⅰ |
| Vpr-R | CAC*CGGCCG*CAGCCGGAGCTGCC | *Eag* Ⅰ |
| YwmD-F | CC*CTCGAG*GTAAGAGAGGGTGAAAAAATTGCTGGCTGC | *Xho* Ⅰ |
| YwmD-R | CAC*CGGCCG*CGGCAAAAGACGGGGAG | *Eag* Ⅰ |
| BslB-F | CC*CTCGAG*GTAAGAGAGGATGTTAAAAAGAACTTCATTTG | *Xho* Ⅰ |
| BslB-R | CAC*CGGCCG*CATGAGCTTGGCCTGAAGG | *Eag* Ⅰ |
| LytF-F | CC*CTCGAG*GTAAGAGAGGCTGAAAAAGAAATTAGCAGCAGG | *Xho* Ⅰ |
| LytF-R | CAC*CGGCCG*CTGCTTCAGCTGGTGTCAC | *Eag* Ⅰ |
|  |  |  |
| amyQ-F | TAGT*GGTACC*GGCGGCGTTCTGTTTC | *Kpn* Ⅰ |
| amyQ-R | TTAC*CTCGAG*TCTTGACACTCCTTATTTGATTTTTTG | *Xho* Ⅰ |
| nprE-F | TAGT*GGTACC*CAGCAGTTCTTTTCCGTCC | *Kpn* Ⅰ |
| nprE-R | TTAC*CTCGAG*AATAAATCCCCCTTTTTGAAAATACTG | *Xho* Ⅰ |
| gsiB-F | TAGT*GGTACC*CTATCGAGACACGTTTGGCTG | *Kpn* Ⅰ |
| gsiB-R | TTAC*CTCGAG*TTTGAATTCCTCCTTTAATTGGTGTTG | *Xho* Ⅰ |
| tufA-F | TAGT*GGTACC*TTGATTTTGCCGCTTAACTC | *Kpn* Ⅰ |
| tufA-R | TTAC*CTCGAG*TCTAAAATCCTCCTTAAGAGCTTT | *Xho* Ⅰ |
| sodA-F | TAGT*GGTACC*GAAATGCTGGCGGCA | *Kpn* Ⅰ |
| sodA-R | TTAC*CTCGAG*GATAATTCCTCCTTAGTATATATGTACTGAAATG | *Xho* Ⅰ |
| gapA-F | TAGT*GGTACC*ATCGAGGCTTACTTTAAAAAGC | *Kpn* Ⅰ |
| gapA-R | TTAC*CTCGAG*GATTGTTTCCTCCTTTAAATAAGTG | *Xho* Ⅰ |
| RBS1-F | A*CTCGAG*GAATGGGAGGATGAAGAAAGCATTTATTTTATCTGC | *Xho* Ⅰ |
| RBS1-R | CCTCCCATTC*CTCGAG*TTTGAATTCCTCCTTTAATTGG | *Xho* Ⅰ |
| RBS2-F | A*CTCGAG*GAGAAGGAGGATGAAGAAAGCATTTATTTTATCTGC | *Xho* Ⅰ |
| RBS2-R | CCTCCTTCTC*CTCGAG*TTTGAATTCCTCCTTTAATTGG | *Xho* Ⅰ |
| RBS3-F | A*CTCGAG*GGATAGGAGGATGAAGAAAGCATTTATTTTATCTGC | *Xho* Ⅰ |
| RBS3-R | CCTCCTATCC*CTCGAG*TTTGAATTCCTCCTTTAATTGG | *Xho* Ⅰ |
| RBS4-F | A*CTCGAG*AGGAGGATGACGTCATGAAGAAAGCATTTATTTTATC | *Xho* Ⅰ |
| RBS4-R | GACGTCATCCTCCT*CTCGAG*TTTGAATTCCTCCTTTAATTGG | *Xho* Ⅰ |
| RBS5-F | A*CTCGAG*GGTGAGTTCATGAAGAAAGCATTTATTTTATCTGC | *Xho* Ⅰ |
| RBS5-R | GAACTCACC*CTCGAG*TTTGAATTCCTCCTTTAATTGG | *Xho* Ⅰ |
| RBS6-F | AAGGAGGTGAAAGGCATGAAGAAAGCATTTATTTTATCTGC |  |
| RBS6-R | GCCTTTCACCTCCTTTCAAAA*CTCGAG*TTTGAATTCCTC | *Xho* Ⅰ |
| RBS7-F | GGAAAGGAGGCATAGAGACATGAAGAAAGCATTTATTTTATC |  |
| RBS7-R | GTCTCTATGCCTCCTTTCC*CTCGAG*TTTGAATTCCTCCTTTAATTG | *Xho* Ⅰ |

* Letters underlined represent restriction sites.

**Table S2.** Signal peptides

| Name | Amino acid sequences | Length |
| --- | --- | --- |
| SP*_aprE_* | VRSKKLWISLLFALTLIFTMAFSNMSAQA | 29 |
| SP*_ydjM_* | MLKKVILAAFILVGSTLGAFSFSSDASA | 28 |
| SP*_yqxL_* | MFKKLLLATSALTFSLSLVLPLDGHAKA | 28 |
| SP*_abnAw_* | MKKKKTWKRFLHFSSAALAAGLIFTSAAPAEA | 32 |
| SP*_ykwD_* | MKKAFILSAAAAVGLFTFGGVQQASA | 26 |
| SP*_dacB_* | MRIFKKAVFVIMISFLIATVNVNTAHA | 27 |
| SP*_nprE_* | MGLGKKLSVAVAASFMSLSISLPGVQA | 27 |
| SP*_wapA_* | MKKRKRRNFKRFIAAFLVLALMISLVPADVLAK | 34 |
| SP*_vpr_* | MKKGIIRFLLVSFVLFFALSTGITGVQAAPA | 31 |
| SP*_ywmD_* | VKKLLAAGIIGLLTVSIASPSFA | 23 |
| SP*_bslB_* | VLKRTSFVSSLFISSAVLLSILLPSGQAHA | 30 |
| SP*_yytF_* | LKKKLAAGLTASAIVGTTLVVTPAEA | 26 |

**Table S3.** RBS sequences

| RBS | Sequence (5’-3’) |
| --- | --- |
| RBS1 | GAATGGGAGG |
| RBS2 | GAGAAGGAGG |
| RBS3 | GGATAGGAGG |
| RBS4 | AGGAGGATGACGTC |
| RBS5 | GGTGAGTTC |
| RBS6 | TTTTGAAAGGAGGTGAAAGGC |
| RBS7 | GGAAAGGAGGCATAGAGAC |

**Figure**

**Figure S1**. Optimal temperature, pH, and specific activity of AmyS and AmySM‌

(a) Optimal temperature of AmySM and AmyS. (b) Optimal pH of AmySM and AmyS. (c) Specific activity of AmySM and AmyS.

**Figure S2**. Structural insights into the enhanced specific activity of the AmySM triple mutant T151A/K178E/T458A. (a) Structural localization of the three mutation sites in *Geobacillus stearothermophilus* amylase (AmyS). AmyS consists of three domains: the catalytic core domain (green), the B-domain (purple), and the C-domain (cyan). T151A and K178E are located within the B-domain, whereas T458A resides in the C-domain. (b) Interaction between docked maltoheptaose and aromatic residues in the B-domain. Three aromatic residues-Y200 at the -4 subsite, W167 at the -5 subsite, and W140 at the -6 subsite-contribute to substrate binding through π–π stacking. (c) Local structural view of the wild-type K178 site. K178 forms a salt bridge with the nearby E129 residue, helping stabilize the local β-sheet structure. (d) Local structural view of the K178E mutation. The K178E substitution disrupts the original salt bridge, increasing the flexibility of this region and inducing a secondary structure change from β-sheet to loop.

**Figure S3**. Structural dynamics and superimposition of WT and M3 variants. (a-b) Root Mean Mean Square Deviation (RMSD) values of the WT (a) and M3 variant (b) over the course of molecular dynamics (MD) simulations. (c) Root Mean Square Fluctuation (RMSF) of each residue in WT (blue) and M3 (red) variants, indicating the flexibility of individual residues. (d) Superimposition of WT and M3 structures. Critical carbon atoms of residues 147, 174, 454, 167 and 140 in WT are displayed as sticks in lime, while the same set of residues in M3 are shown as sticks in aquamarine. An arrow indicates that the loop in M3 is closer to the substrate relative to that in WT.

**Figure S4**. Effects of selected optimal signal peptides on α-amylase secretion in *B. subtilis* 168 via shake-flask fermentation. Extracellular α-amylase activity of the control strain *B. subtilis* 168 SM was defined as 100%. Activities of derived strains were expressed as relative percentages (±SD).
