# Supplementary figures and images for: Synergistic strategies for high production of Geobacillus stearothermophilus α-amylase in Bacillus subtilis
Source: J Ind Microbiol Biotechnol. 2025 Dec 19;53:kuaf036. doi: 10.1093/jimb/kuaf036 (PMC12784944; doi:10.1093/jimb/kuaf036)

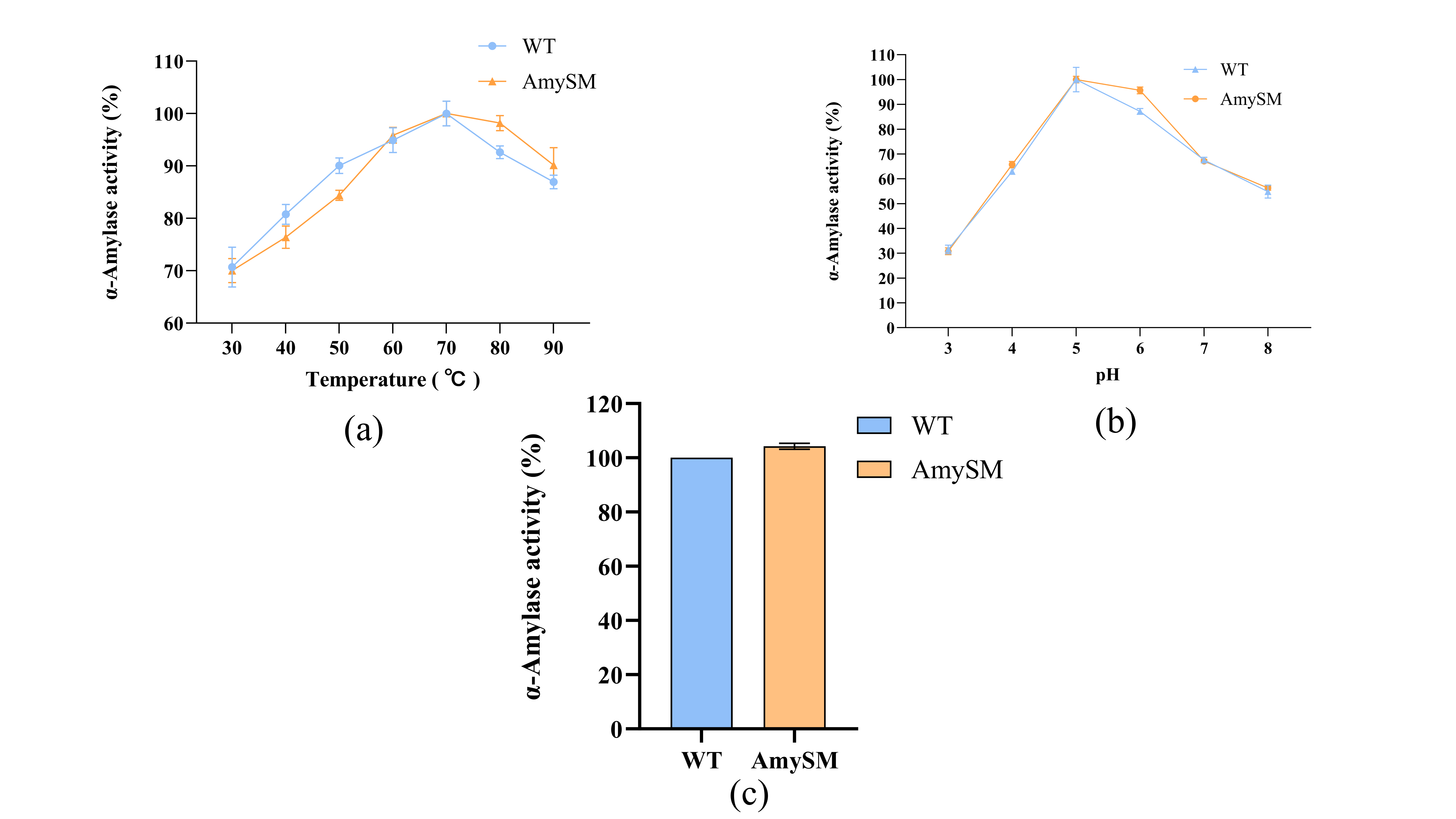

Supplement: kuaf036_Supplemental_Files [file kuaf036_supplemental_files.zip › Figure S1.TIF]

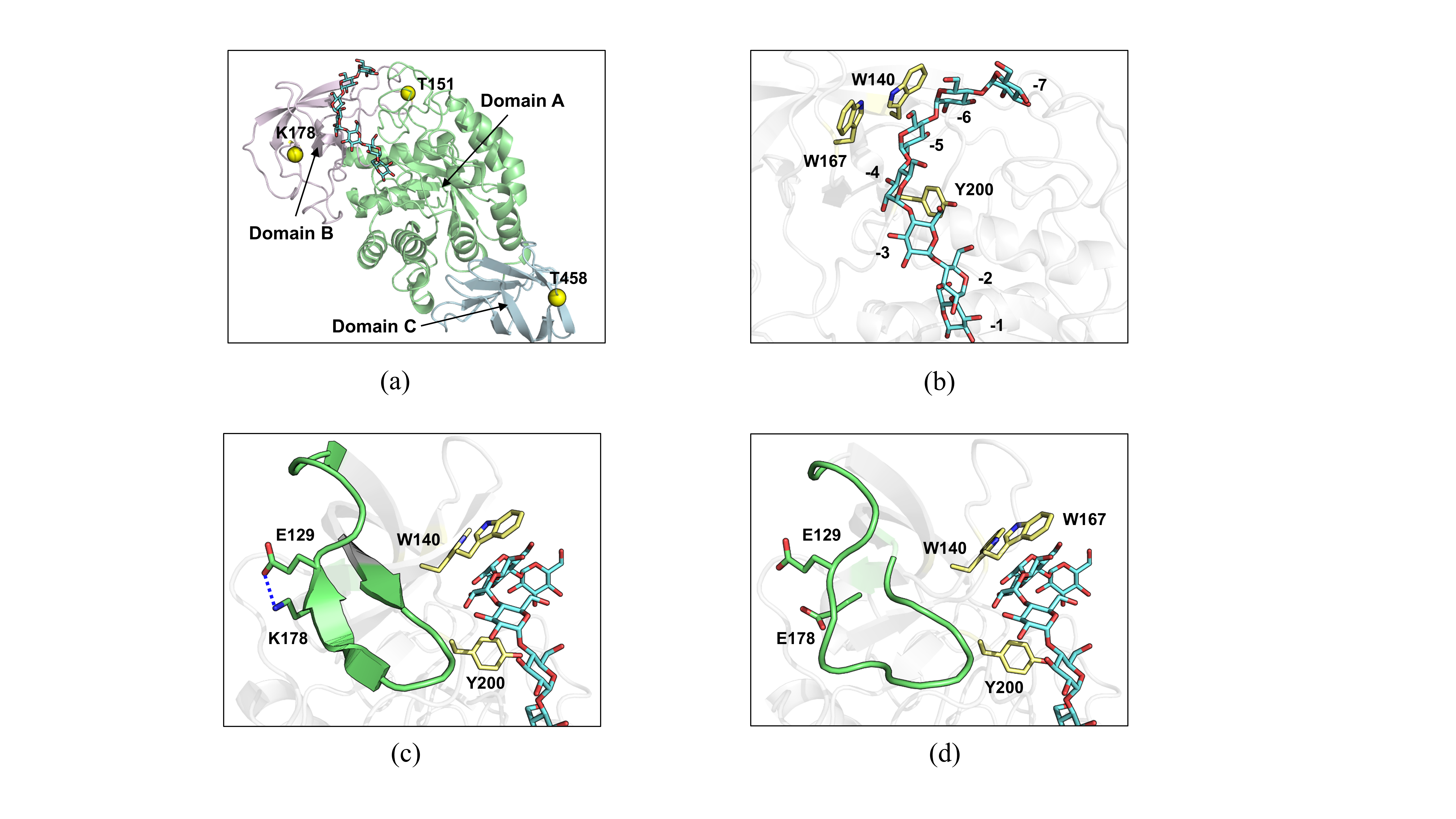

Supplement: kuaf036_Supplemental_Files [file kuaf036_supplemental_files.zip › Figure S2.tif]

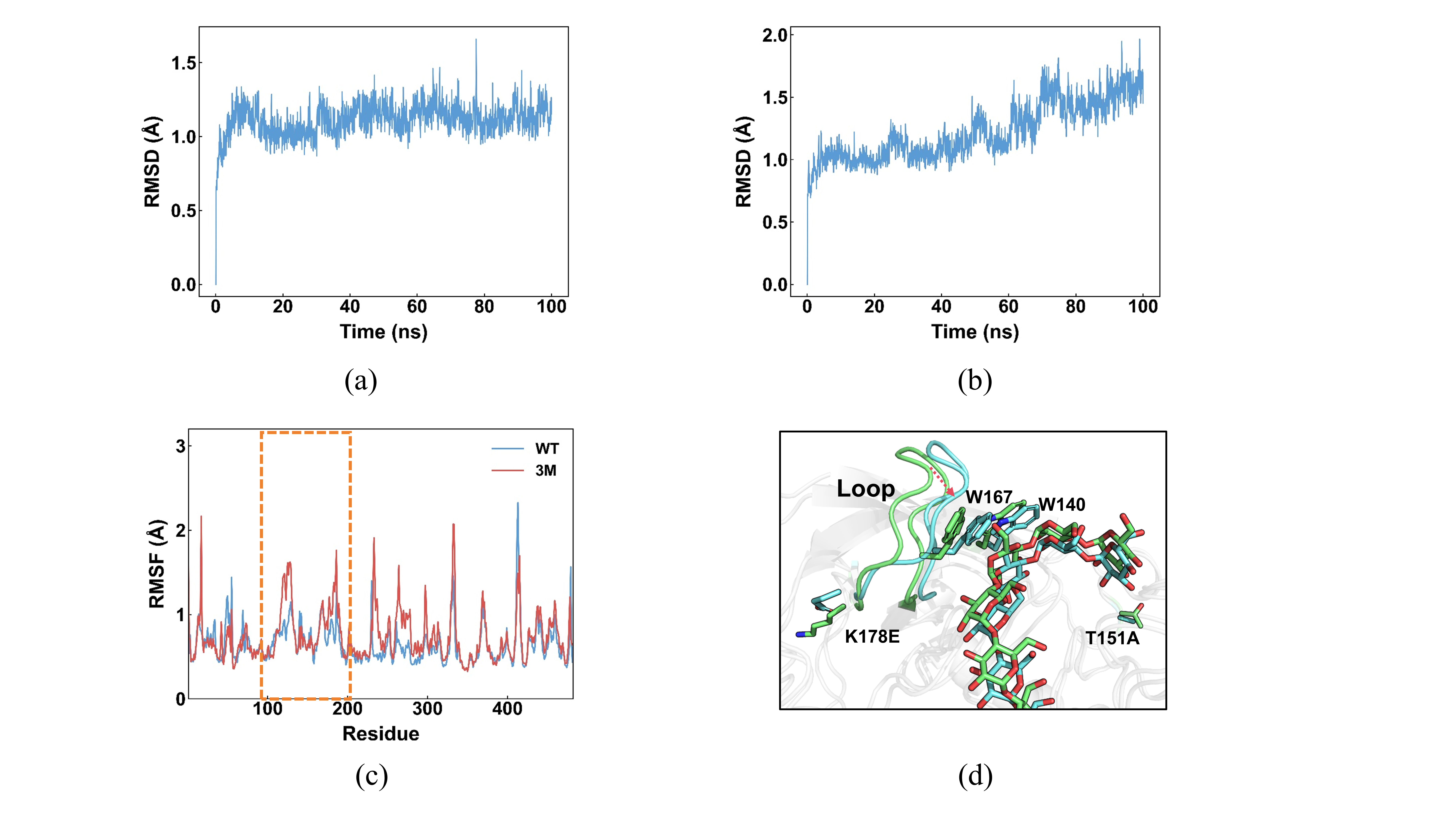

Supplement: kuaf036_Supplemental_Files [file kuaf036_supplemental_files.zip › Figure S3.tif]

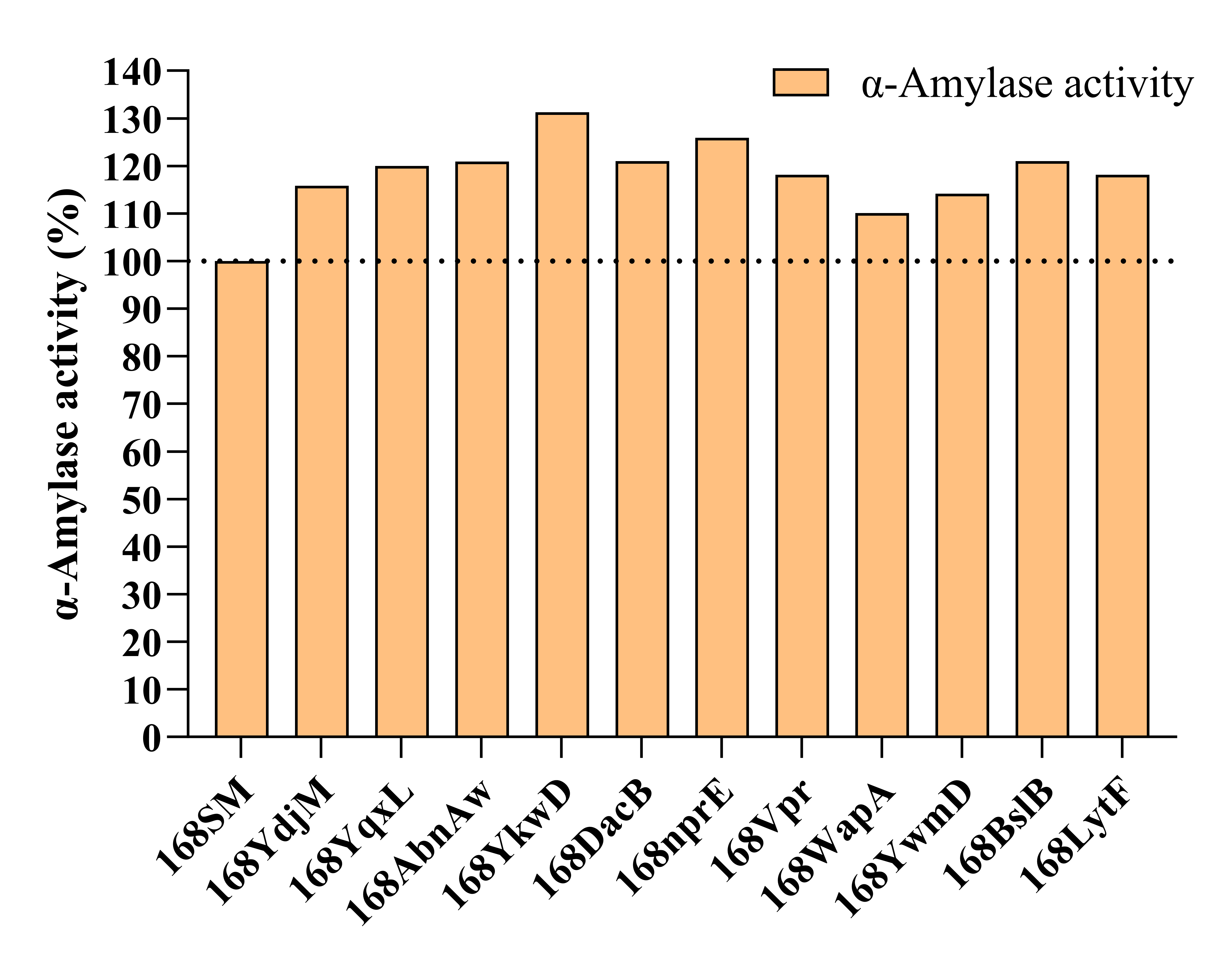

Supplement: kuaf036_Supplemental_Files [file kuaf036_supplemental_files.zip › Figure S4.tif]
